# Anti‐β2GPI/β2GPI induces human neutrophils to generate NETs by relying on ROS

**DOI:** 10.1002/cbf.3363

**Published:** 2019-01-30

**Authors:** Yanqiu You, Yanhong Liu, Fujun Li, Fengyun Mu, Caijun Zha

**Affiliations:** ^1^ Clinical Laboratory The Second Affiliated Hospital of Harbin Medical University Harbin China; ^2^ Department of Anesthesiology The First Affiliated Hospital of Harbin Medical University Harbin China

**Keywords:** anti‐β_2_GPI/β_2_GPI, neutrophil extracellular traps, primary human leukocytes, propidium iodide, reactive oxygen species

## Abstract

Neutrophils participate in the regulation of pathogens by phagocytosis as well as by generating neutrophil extracellular traps (NETs). Antiphospholipid antibodies, particularly those targeting beta‐2‐glycoprotein I (β2GPI), stimulate monocytes, platelets, and endothelial cells with prothrombotic participation. This study aimed to explore NET generation in response to anti‐β2GPI/β2GPI. A series of experiments involving the separation of primary human leukocytes, NETosis quantification using propidium iodide, exploration of NETosis by fluorescence microscopy, western blotting, examination of free Zn^2+^ using FluoZin‐3, and reactive oxygen species (ROS) examination with dihydrorhodamine 123 were performed in this study. We found that anti‐β2GPI/β2GPI triggered NETosis, resembling phorbol 12‐myristate 13‐acetate (PMA)‐induced NETosis in magnitude and morphology. The anti‐β_2_GPI/β_2_GPI complex in isolation stimulated NETs without relying on p38, protein kinase B (AKT), extracellular signal‐related kinase (ERK) 1/2, and zinc signals. NET generation was unaffected by the NADPH oxidase suppressor DP1. The anti‐β_2_GPI/β_2_GPI complex stimulated ROS generation without relying on NADPH oxidase, which may participate in NET generation triggered via the anti‐β_2_GPI/β_2_GPI complex. In summary, our results indicate that the anti‐β_2_GPI/β_2_GPI complex reinforced NET generation by relying on ROS.

**The significance of the paper in the context of current knowledge:**

Neutrophils as one of the first lines of defence and essential in the response to pathogen invasion. They eradicate bacteria via phagocytosis or by releasing antimicrobial proteins in degranulation. In this study, we explored the capability of anti‐β_2_GPI/β_2_GPI to stimulate NETosis, demonstrating that anti‐β_2_GPI/β_2_GPI is a promising method for triggering NET. Anti‐β_2_GPI/β_2_GPI induced ROS generation without relying on NADPH oxidase, which contributes to NETosis independently of ERK1/2, Zn^2+^, or AKT. Our results showed that anti‐β2GPI/β2GPI triggered NETosis, resembling PMA‐induced NETosis in magnitude as well as morphology. The anti‐β_2_GPI/β_2_GPI complex in isolation stimulated NETs without relying on p38, AKT, ERK1/2, or zinc signals. The anti‐β_2_GPI/β_2_GPI complex stimulated ROS generation without relying on NADPH oxidase, which may participate in NET generation triggered via the anti‐β_2_GPI/β_2_GPI complex.

## 1 INTRODUCTION

Neutrophils represent the most abundant cells in the immune system and are responsible for 50‐70% of leucocytes in human blood.^1^ As one of the first lines of defence, they are essential in the response to pathogen invasion.^2^ They eradicate bacteria via phagocytosis or by releasing antimicrobial proteins in degranulation.^3^ Neutrophils receive an innovative pattern of apoptosis programing signals known as NETosis that induces the release of neutrophil extracellular traps (NETs), which are composed of double‐stranded DNA resembling a net with a histone coating as well as antimicrobial agents including myeloperoxidase.^4^, ^5^ NETs attract and eradicate bacteria and counteract viruses.^6^ NETosis occurs in reaction to multiple fungal as well as bacterial pathogens. Initially, phorbol 12‐myristate 13‐acetate (PMA) stimulates NETosis.^7^ As a stimulator of protein kinase C (PKC), PMA stimulates several downstream pathways triggering NADPH oxidase, which generates the reactive oxygen species (ROS) necessary for NETosis.^8^

NETs are related to multiple autoimmune disorders such as small‐vessel vasculitis, rheumatoid arthritis, psoriasis, and systemic lupus erythematosus (SLE).^9^ Consequently, the release of chromatin during the generation of NETs is a source of autoantigens.^10^ If NETs are produced during viral infections, they are particularly effective in influencing tolerance and inducing autoimmunity, because viruses trigger the delivery of inflammatory cytokines including Type I interferon, which participates in SLE.^11^

Antiphospholipid antibodies identify not only thrombin, but also β2GPI.^12^, ^13^ Anti‐β2GPI antibodies are commonly used for clinical assays.^14^ The understanding of reactions downstream of β2GPI is more detailed. As a cationic protein that binds to lipids, β2GPI shows elevated concentrations in blood and can be generated via the liver, monocytes, endothelial cells, and trophoblasts.^15^ Although several studies have suggested that β2GPI affects NET delivery,^16^ its aetiology is unclear.

This study was conducted to explore the capability of anti‐β_2_GPI/β_2_GPI to stimulate NETosis, demonstrating that anti‐β_2_GPI/β_2_GPI is a promising method for triggering NETs. Anti‐β_2_GPI/β_2_GPI induces ROS generation without relying on NADPH oxidase, which contributes to NETosis independently of extracellular signal‐related kinase (ERK) 1/2, Zn^2+^, and AKT.

## 2 MATERIALS AND METHODS

### 2.1 Separation of primary human leukocytes (PHL)

PHL including monocytes, lymphocytes, and granulocytes were separated from blood subjected to heparinization and obtained from healthy participants. Participants were maily women (only one man) 22‐42 years of age. Subjects who smoked, drank more than a moderate quantity of alcohol, displayed contemporary infection, and had a correlated clinical history were excluded. Fully informed consent was acquired from participants. To separate white blood cells (WBCs), 6% hydroxyethyl starch solution was added in a 2‐fold volume to the blood. Phosphate‐buffered saline (PBS) was used to wash the cells twice before sedimentation, which was conducted at room temperature (RT) for 45‐60 min. Hypotonic lysis was carried out utilizing the remaining red blood cells. Ethical approval was acquired from the institutional ethics review board of The First Affiliated Hospital of Harbin Medical University.

### 2.2 NETosis quantification using propidium iodide (PI)

NET generation was quantified by examining increased fluorescence emissions from PI in response to DNA binding outside of the cells. Cells were plated in 96‐well plates in the examination buffer. After 2‐4 h of incubation at 37°C, PI was added at 10 mg/mL. A Tecan Ultra 384 fluorescence well plate reader (Männedorf, Switzerland) was utilized to determine fluorescence levels at an excitation wavelength of 360 nm and emission wavelength of 612 nm.

### 2.3 Exploration of NETosis using fluorescence microscopy

Anti‐β_2_GPI/β_2_GPI or PMA was applied to stimulate the cells at 37°C in cultivation media. SYTOX green was added at 1 μM followed by incubation for 4 h. Cells were placed on glass slides after centrifugation in a cytospin at 300 × g for 5 min. A Zeiss Axioskop was utilized to observe fluorescence (Oberkochen, Germany). A Nikon Coolpix 4500 digital camera was used to capture the images at 10X magnification (Tokyo, Japan).

### 2.4 Western blotting (WB)

The cell lysate was electrophoresed in 10% sodium dodecyl sulfate‐polyacrylamide gel electrophoresis gels and transferred onto a polyvinylidene difluoride membrane (Bio‐Rad, Hercules, CA, USA). The membrane was blocked in 3% dry nonfat milk in saline/0.05% Tween‐20 (TBST) for 1 h at RT, washed with TBST three times, and then incubated with primary antibodies against p‐AKT, AKT, p‐p38, p38, p‐ERK1/2, ERK1/2, and β‐actin (Cell Signalling Technology, Danvers, MA, USA) overnight at 4°C. Following three washes with TBST, the membranes were incubated with horseradish peroxidase‐conjugated secondary antibodies for 1 h at RT. Finally, the western blotting (WB) signals were developed using ECL detection reagents (Millipore, Billerica, MA, USA).

### 2.5 Examination of free Zn^2+^ using FluoZin‐3

Free Zn^2+^ inside the cells was examined as previously described.^17^ Briefly, WBCs were treated for 1 h with anti‐β2GPI/β2GPI in measuring buffer at 37°C. During the final 30 min, FluoZin‐3 acetoxymethyl ester was added to the cells. PBS was used to wash the cells, which were evaluated by flow cytometry using a BD FACSCalibur flow cytometer (BD Biosciences, Franklin Lakes, NJ, USA). FluoZin‐3 emission was evaluated in FL‐1. Side and forward scatter were measured to differentiate lymphocytes, monocytes, and granulocytes. The levels of free Zn^2+^ were evaluated as the average intensities (fluorescence) of every cell population using a separation constant in terms of the Zn^2+^/FluoZin‐3 complex (8.9 nM), determining the lowest intensity (fluorescence) with N,N,N′,N′‐tetrakis (2‐pyridylmethyl) ethylenediamine (TPEN, 50 μM) and highest fluorescence intensity with the Zn^2+^ ionophore pyrithione.

### 2.6 ROS examination with DHR123

For ROS examination, cells were subjected to 30‐min loading using 1 μg/mL (dihydrorhodamine) (DHR) 123 as a loading buffer (measuring buffer with bovine serum albumin [*w*/*v*; 0.3%]) at 37°C. Measuring buffer was used to wash the cells twice, which were subsequently added to 96‐well plates. Fluorescence was measured at the initial stage of the experiment and after 45 min at 37°C using a fluorescence plate reader. The excitation and emission wavelengths were 485 and 535 nm, respectively.

### 2.7 Statistical analysis

Data are presented as the mean ± SD unless otherwise indicated. The data were analysed by Student *t* test or a one‐way analysis of variance using Prism version 6 (GraphPad, Inc., La Jolla, CA, USA). Values of *P* < 0.05 were considered significant.

## 3 RESULTS

### 3.1 Induction of NETs by anti‐β2GPI/β2GPI

**Figure 1:**
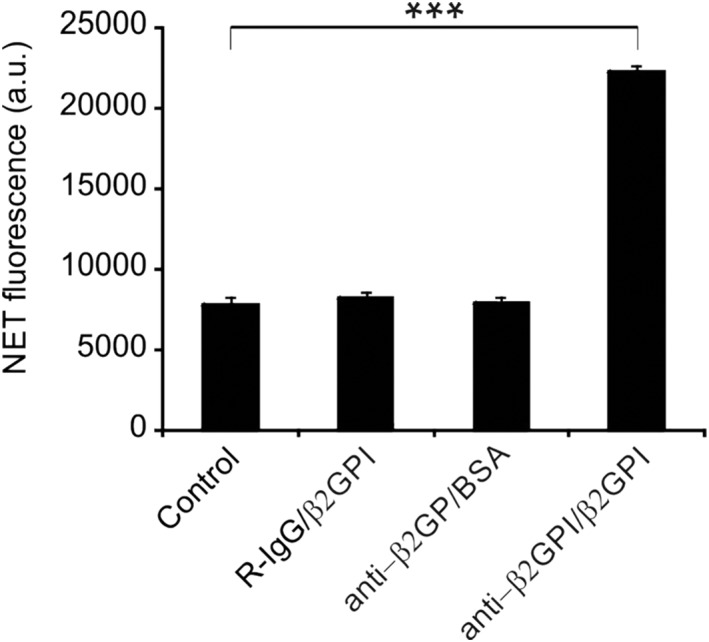
Induction of NETosis by anti‐β2GPI/β2GPI. Primary human leukocytes were treated with anti‐β2GPI/β2GPI complex, isotype control for 4 h at 37°C. extracellular NET‐DNA was quantified. Data are presented as the mean ± SD of three independent experiments. ***, *P* < 0.001

**Figure 2:**
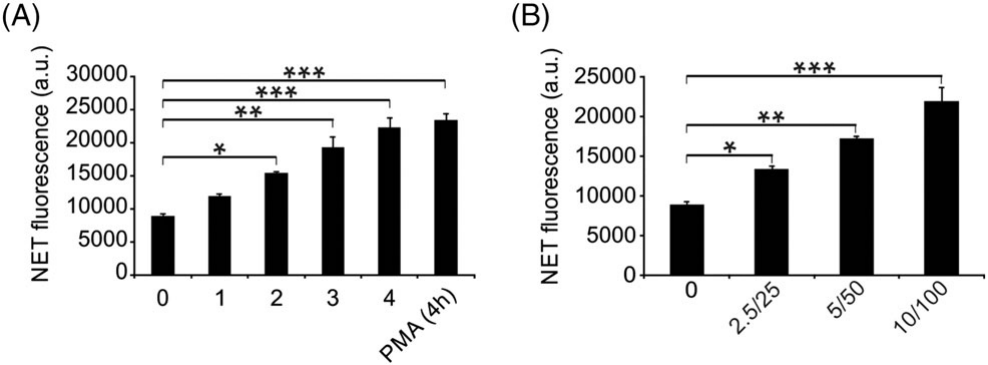
Induction of NETosis by anti‐β2GPI/β2GPI. A, Primary human leukocytes (PHL) were treated with anti‐β2GPI/β2GPI for 4 h at 37°C and extracellular NET‐DNA was quantified. B, PHL were treated with anti‐β2GPI/β2GPI at the indicated concentration for 4 h at 37°C and extracellular NET‐DNA was quantified. Data are presented as the mean ± SD of three independent experiments. *P < 0.05, **P < 0.01, ***P < 0.001

**Figure 3:**
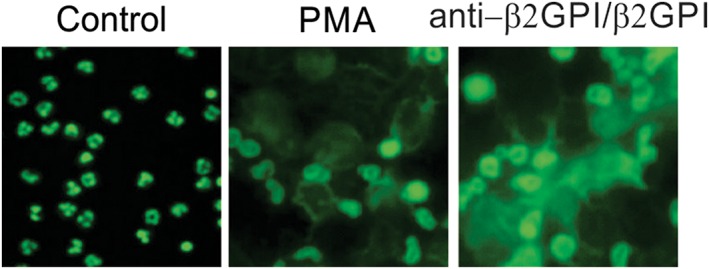
NETs induced by PMA and anti‐β2GPI/β2GPI. Fluorescence microscopy images of leukocytes stained with SYTOX green

NETosis stimulation was carried out via various mercury species in PHL supplemented with anti‐β2GPI (10 μg/mL)/β2GPI (100 μg/mL). NET generation was quantified by examining the DNA outside the cells by PI staining (Figure 1). Fluorescence was noticeably increased with anti‐β2GPI/β2GPI, suggesting the generation of NETs. Independent supplementation with β2GPI or anti‐β2GPI did not affect the fluorescence. Additional procedures were conducted using anti‐β2GPI/β2GPI. The effect of anti‐β2GPI/β2GPI was dependent on the time and concentration and showed a similar effect as PMA, a known stimulator of NETosis (Figure 2). Anti‐β2GPI/β2GPI‐induced NETs were confirmed by SYTOXgreen staining (Figure 3). Briefly, anti‐β2GPI/β2GPI triggered NETosis resembling PMA‐induced NETosis in magnitude and morphology.

### 3.2 Kinase phosphorylation

**Figure 4:**
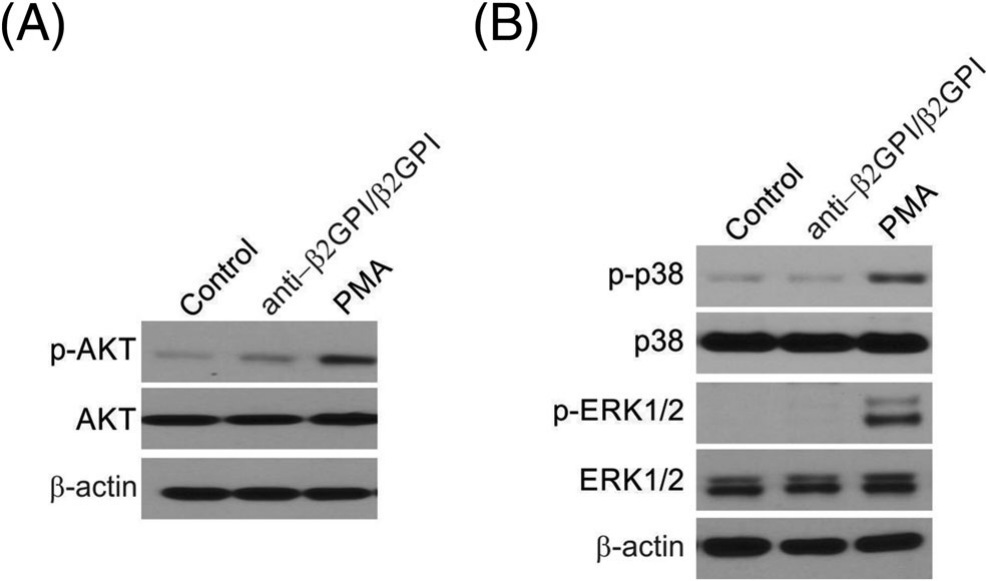
Role of kinases in anti‐β2GPI/β2GPI‐induced NETosis. Leukocytes were incubated with PMA or anti‐β2GPI/β2GPI for 30 min. Western blot analysis was performed using antibodies against p‐AKT (Ser473) A, and (ex) p‐p38 MAPKs and ERK1/2 B,

In order to examine the aetiology of how anti‐β2GPI/β2GPI triggered NETosis, WB was applied to explore AKT function with the help of antibodies counteracting AKT serine phosphorylation (Figure 4A). PMA promoted phosphorylation in some proteins.^18^ However, anti‐β2GPI/β2GPI was unable to do so. Moreover, phosphorylation of ERK1/2 and p38 MAPK was reinforced via PMA but not with anti‐β2GPI/β2GPI (Figure 4B), suggesting that anti‐β2GPI/β2GPI triggered NETosis without relying on stimulation of p38, ERK1/2, or AKT signalling pathway.

### 3.3 Zn^2+^ delivery

**Figure 5:**
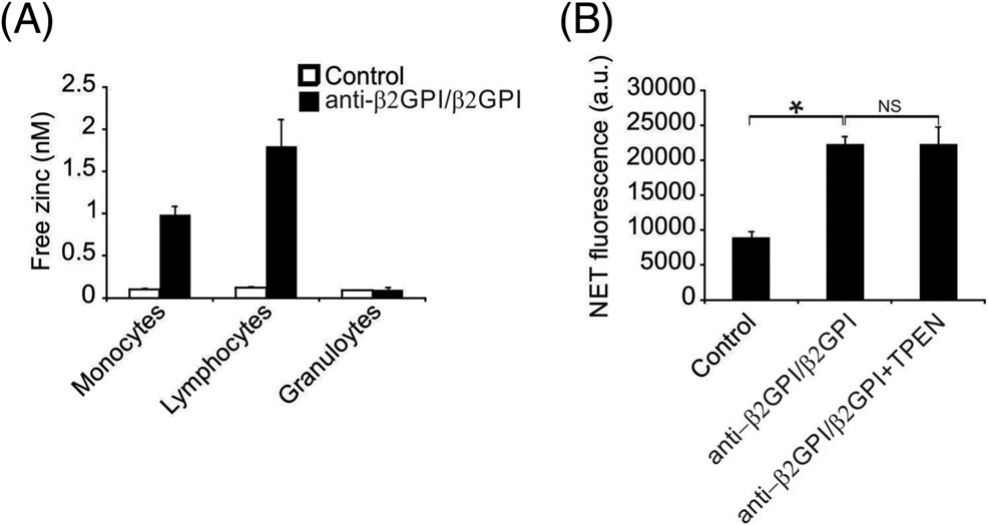
Role of Zn^2+^ in anti‐β2GPI/β2GPI‐induced NETosis. A, Leukocytes were treated with anti‐β2GPI/β2GPI for 1 h, followed by loading with Fluo‐Zin‐3. Zinc‐dependent fluorescence was measured. B, Leukocytes were pre‐treated with TPEN, followed by incubation with anti‐β2GPI/β2GPI for 4 h. extracellular NET‐DNA was quantified. Data are presented as the mean ± SD from three independent experiments. *, *P* < 0.05

Zn^2+^ delivery was reinforced in lymphocytes and monocytes in response to anti‐β2GPI/β2GPI rather than in WBC granulocytes (Figure 5A). Chelation of free Zn^2+^ inside the cells using TPEN, a Zn^2+^‐selective chelator that can penetrate membranes, failed to influence NETosis triggered by anti‐β2GPI/β2GPI (Figure 5B). This indicates that Zn^2+^ delivery did not participate in NETosis triggered by anti‐β2GPI/β2GPI.

### 3.4 ROS

**Figure 6:**
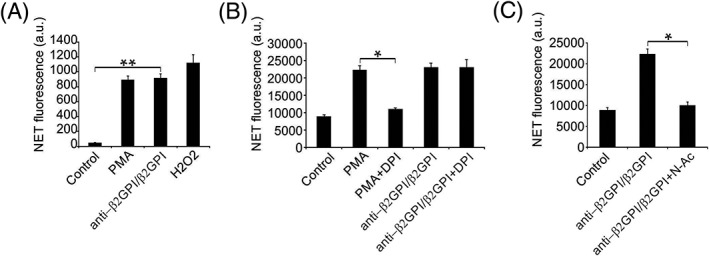
Effect of NADPH oxidase and ROS in anti‐β2GPI/β2GPI‐induced NETosis. A, Leukocytes were loaded with the profluorophore DHR123, followed by measuring the increase in fluorescence after incubation with PMA and anti‐β2GPI/β2GPI. B‐C, leukocytes were pre‐incubated with DPI and *N*‐acetylcysteine for 10 min, followed by treatment with PMA (50 ng/mL). Extracellular NET‐DNA was quantified. Data are presented as the mean ± SD of three independent experiments. *, *P* < 0.05; **, *P* < 0.01

A previous study showed that ROS generation is crucial in NETosis.^10^ The pro‐fluorophore DHR123, which is sensitive to redox, displayed similar fluorescence subsequent to supplementation with anti‐β2GPI/β2GPI and PMA, despite its weakness compared with the strongest activation by H_2_O_2_ (Figure 6A). The NADPH oxidase inhibitor diphenylene iodonium (DPI) notably suppressed NETosis triggered via PMA, but not in the presence of anti‐β2GPI/β2GPI (Figure 6B). *N*‐Acetylcysteine counteracted oxidation and suppressed NETosis triggered via anti‐β2GPI/β2GPI, indicating the influence of ROS on these reactions (Figure 6C). Our findings indicate that anti‐β2GPI/β2GPI stimulated NETosis by activating ROS generation independently of NADPH oxidase.

## 4 DISCUSSION

NETosis is a crucial reaction used by WBCs to eliminate microorganisms.^19^ It affects both adaptive and innate immune reactions and is crucial for autoimmune reactions in disorders including SLE.^20^ Although numerous studies have identified reactions linked with NET delivery, the understanding of the reactions inside cells that participate in NET generation is insufficient regarding the various NET stimulators.

Although numerous candidate targets of anti‐β_2_GPI/β_2_GPI have been identified in signal pathways to cause NETosis, most promising mechanisms were shown to be irrelevant to NETosis.^12^, ^13^ PKC is a crucial kinase in pathways inducing NETosis and is stimulated by anti‐β_2_GPI/β_2_GPI, likely by increasing the free Ca^2+^ concentration inside cells.^21^, ^22^ NETosis stimulated via the PKC stimulator, PMA leads to phosphorylation of PKC, p38 MAPKs, and ERK1/2.^23^, ^24^ However, no stimulation of these kinases was detected in the reaction to anti‐β2GPI/β2GPI. Zn^2+^ is crucial for NETosis triggered via PMA.^25^ Evaluation of free Zn^2+^ in the reaction to anti‐β_2_GPI/β_2_GPI revealed noticeably increased concentrations of lymphocytes and monocytes, which was similar to the results of previous studies.^26^ Zinc functions downstream of ROS and is crucial but insufficient for NETosis.^17^, ^27^ Consequently, anti‐β_2_GPI/β_2_GPI replaced Zn^2+^ with ROS, eliminating the requirement for Zn^2+^.

NETosis is associated with the stimulation of NADPH oxidase, which generates ROS crucial for NET generation.^28^ Several studies showed that NADPH oxidase does not always participate in NETosis and relies on other sources of ROS.^29^, ^30^ However, the pro‐fluorophore DHR123, which is sensitive to oxidation, displayed similar fluorescence subsequent to activation by PMA and anti‐β_2_GPI/β_2_GPI, indicating similar ROS generation, although they arose from different sources. Notably, mercurial compounds alter the process of ROS arising from NADPH oxidase. Not only organic but also inorganic patterns of mercury cause mitochondrial injury following ROS delivery.

In conclusion, our study revealed that anti‐β_2_GPI/β_2_GPI treatment is a promising method for stimulating NETosis through reactions relying on ROS.

## CONFLICT OF INTEREST

none

## FUNDING INFORMATION

This work was supported by research fund of Heilongjiang Provincial Health and Family Planning Commission [Grant number 2016‐070].
